# Immunoinformatic design of a COVID-19 subunit vaccine using entire structural immunogenic epitopes of SARS-CoV-2

**DOI:** 10.1038/s41598-020-77547-4

**Published:** 2020-11-30

**Authors:** Esmaeil Behmard, Bijan Soleymani, Ali Najafi, Ebrahim Barzegari

**Affiliations:** 1 Medical Biology Research Center, Health Technology Institute, Kermanshah University of Medical Sciences, Zakariya Razi Blvd., Kermanshah, Iran; 2 Pharmaceutical Sciences Research Center, Shiraz University of Medical Sciences, Shiraz, Iran; 3 Molecular Biology Research Center, Systems Biology and Poisonings Institute, Baqiyatallah University of Medical Sciences, Tehran, Iran

**Keywords:** Viral infection, Protein vaccines, Molecular modelling, Computational models

## Abstract

Coronavirus disease 2019 (COVID-19) is an acute pneumonic disease, with no prophylactic or specific therapeutical solution. Effective and rapid countermeasure against the spread of the disease’s associated virus, SARS-CoV-2, requires to incorporate the computational approach. In this study, we employed various immunoinformatics tools to design a multi-epitope vaccine polypeptide with the highest potential for activating the human immune system against SARS-CoV-2. The initial epitope set was extracted from the whole set of viral structural proteins. Potential non-toxic and non-allergenic T-cell and B-cell binding and cytokine inducing epitopes were then identified through a priori prediction. Selected epitopes were bound to each other with appropriate linkers, followed by appending a suitable adjuvant to increase the immunogenicity of the vaccine polypeptide. Molecular modelling of the 3D structure of the vaccine construct, docking, molecular dynamics simulations and free energy calculations confirmed that the vaccine peptide had high affinity for Toll-like receptor 3 binding, and that the vaccine-receptor complex was highly stable. As our vaccine polypeptide design captures the advantages of structural epitopes and simultaneously integrates precautions to avoid relevant side effects, it is suggested to be promising for elicitation of an effective and safe immune response against SARS-CoV-2 in vivo.

## Introduction

Coronaviruses (CoVs) named for the crown-like spikes on their surface are grouped into four main types, known as α, β, γ and δ^1^. In recent decades, new human β-CoVs have evolved from animal-infecting types, leading to intercontinental pneumonic outbreaks. Severe acute respiratory syndrome coronavirus (SARS-CoV) and Middle East respiratory syndrome coronavirus (MERS-CoV) are two highlight examples, which engendered epidemics in numerous countries worldwide in 2002 and 2012, respectively. SARS-CoV-2 was a return of coronaviruses in December 2019, and is the etiologic agent for the ongoing coronavirus disease 2019 (COVID-19). The viral spill-over originated from a Wuhan seafood market, the place where horseshoe bat SARS-like coronavirus was believed to transmit to humans. By 21st September 2020, over 30.6 million cases and 950,000 deaths have been reported to World Health Organization^2^.

Identifying an appropriate preventive, i.e. a COVID-19 vaccine, is critical for apt response to the SARS-CoV-2 mass contagion. There are diverse candidate types for immunogen development, including but not limited to live-attenuated or inactivated whole virus, DNA vaccines, vectored vaccines, subunit vaccines, and self-assembling virus-like particle vaccines, each with particular advantages and disadvantages. Vaccines based on chemically inactivated CoV virions have led to production of neutralizing antibodies with different levels of protection, but they raise potential side effects and biosafety concerns^3^. DNA vaccines expressing full-length spike protein or its fragments, as well as DNA priming coupled with protein boosting, have also been effective against MERS-CoV infection^4,5^. By intracellular expression of immunogenic antigens, vectored vaccines allow activation of cellular immune response in addition to humoral immunity; they have a proven safety record, however these vectors are limited to presenting one or a reduced number of CoV antigens to the immune system^6^. Subunit vaccines based on recombinant spike protein have been shown to be very well suited for creating immunogenicity against SARS and MERS^7,8^. While DNA, vectored and subunit candidates presenting viral epitopes can elicit a focused antibody response, their subviral components may not portray the full antigenic complexity of the virus, resulting in limited protective efficacy or immunopathology due to unbalanced immune responses^6^. One promising option to confront this issue is the subunit vaccines harbouring numerous and diverse antigenic elements, allowing to produce an inclusive spectrum of native viral antigens.

The process of vaccine development takes at least ten years from bench research to approved vaccine use^9^. Identification of apt elements for developing vaccine immunogens can be promoted using chemistry and topology. As a current trend, this is becoming more of a rational design exercise, which may considerably reduce both the time and costs required for vaccine development and production^10^. In this study, it was hypothesized that advanced computational screening could identify suitable peptides for the construction of a subunit vaccine. We used the full set of the putative structural proteins from SARS-CoV-2 as the substance for extracting antigenic elements creating both B-cell and T-cell immunity. By integrating these peptides together, we developed a multi-epitope-based vaccine polypeptide with appropriate binding to Toll-like receptor 3 (TLR-3) to elicit an effective immune response, and with least possible toxicity and hypersensitivity. We also considered incorporating an adjuvant for augmenting the host antigen-specific immune response. As this method has been validated experimentally with designs against other pathogenic species^11^, we suggest that the proposed structural formulation would be potential to generate an effective immune response against the novel virus. The wet lab researchers are expected to validate our design, hoping to reach protection for the healthy community against the COVID-19 pandemic.

## Results

**Figure 1:**
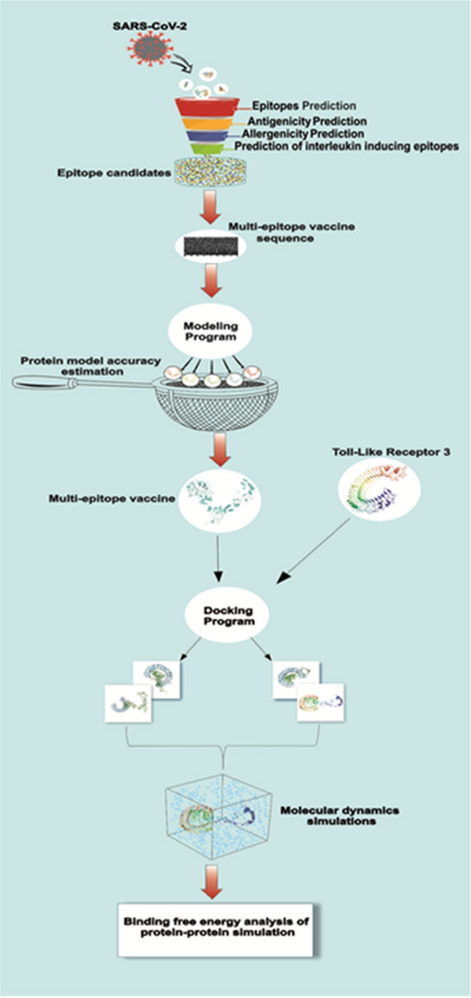
Systemic flowchart of the multi-epitope subunit vaccine building against COVID-19.

The basic steps of the procedure for designing the multi-epitope vaccine are shown in Fig. 1.

### Structural T-cell and B-cell epitopes of SARS-CoV-2

Final selected cytotoxic T lymphocyte (CTL) antigenic epitopes harboured in the structural proteins of SARS-CoV-2 are listed in Table 1, indicating the epitopes for spike glycoprotein (S), envelope protein (E), membrane protein (M) and nucleocapsid phosphoprotein (N). The full sets of predicted epitopes and their properties can be found in Supplementary Tables S1–S4 online, for S, E, M and N proteins, respectively. The IEDB MHC-II prediction tool was applied to predict helper T lymphocyte (HTL) (15-mer) epitopes and their MHC-II binding, results of which are shown in Table 2, and with more detail in Supplementary Table S5 online. Both Tables 1 and 2 also indicate the HLA restriction of the single predicted epitopes. The peptide DVSLVKPSFYVYSRVK contained in the E protein was identified as the only linear B lymphocyte (LBL) epitope, when applying the score value > 0.75 as the criterion for selection (More detail in Supplementary Table S6 online). Using appropriate server tools, antigenic, non-toxic and non-allergenic T-cell or B-cell epitopes that were able to induce interferon-gamma (IFN-γ), interleukin-4 (IL-4) and interleukin-10 (IL-10) cytokines were selected for the design of a multi-epitope vaccine.

**Table 1:** The final set of cytotoxic T lymphocyte (CTL) epitopes selected for multi-epitope vaccine construction.

Protein	Peptide	IC_50_	Antigenicity	Allele
S	^386^KLNDLCFTNV^395^	6.22	2.6927	HLA-A*02:03; HLA-A*02:01
S	^329^FPNITNLCPF^338^	8.63	1.3964	HLA-B*53:01; HLA-B*35:01
S	^200^FKIYSKHTPI^209^	9.13	1.016	HLA-A*02:03
S	^1060^VVFLHVTYV^1068^	13.02	1.5122	HLA-A*02:03; HLA-A*02:06; HLA-A*02:01
S	^587^ITPCSFGGV^595^	20.32	1.3871	HLA-A*68:02; HLA-A*02:06
S	^1207^EQYIKWPWYI^1216^	21.01	1.1122	HLA-A*23:01; HLA-A*24:02
S	^265^YYVGYLQPR^273^	21.9	1.4692	HLA-A*33:01
S	^229^LPIGINITRF^238^	25.21	1.3027	HLA-B*53:01; HLA-B*35:01
S	^512^VLSFELLHA^520^	33.91	1.0776	HLA-A*02:03
S	^408^RQIAPGQTGK^417^	38	1.7893	HLA-A*03:01
S	^1196^SLIDLQELGK^1205^	38.67	1.0275	HLA-A*11:01
S	^644^QTRAGCLIGA^653^	39.78	1.3933	HLA-A*68:02
E	^61^RVKNLNSSR^69^	4.68	0.8998	HLA-A*31:01; HLA-A*30:01
E	^18^LLFLAFVVF^26^	8.32	0.8144	HLA-B*15:01
E	^57^YVYSRVKNL^65^	15.74	0.702	HLA-A*02:03
E	^29^VTLAILTALR^38^	26.92	0.8404	HLA-A*68:01
E	^23^FVVFLLVTL^31^	27.7	0.7403	HLA-A*02:06
E	^26^FLLVTLAIL^34^	39.95	0.9645	HLA-A*02:01
E	^45^NIVNVSLVK^53^	46.43	0.931	HLA-A*68:01
M	^19^QWNLVIGFLF^28^	12.03	1.2302	HLA-A*23:01
M	^12^IAMACLVGL^20^	13.64	1.1306	HLA-A*68:02
M	^6^GTITVEELK^14^	18.08	1.0976	HLA-A*68:01; HLA-A*11:01
M	^35^RTRSMWSFNP^44^	18.67	1.591	HLA-A*30:01
M	^51^SGFAAYSRYR^60^	24.22	1.0034	HLA-A*31:01
M	^22^LVIGFLFLT^30^	25.12	1.2619	HLA-A*02:06
M	^26^FLFLTWICL^34^	32.26	1.4835	HLA-A*02:01
M	^10^IAIAMACLV^18^	34.16	1.1704	HLA-A*02:06
M	^60^VTLACFVLAAV^70^	40.96	1.3368	HLA-A*02:03
M	^29^SFRLFARTR^37^	41.19	0.7038	HLA-A*31:01; HLA-A*33:01
N	^315^FGMSRIGMEV^324^	7.16	0.88	HLA-A*02:03; HLA-A*02:01
N	^361^KTFPPTEPKK^370^	11.43	0.7657	HLA-A*30:01; HLA-A*11:01
N	^100^KMKDLSPR^107^	13.15	1.7575	HLA-A*31:01
N	^193^SSRNSTPGS^201^	32.96	1.2424	HLA-A*30:01
N	^104^LSPRWYFYYL^113^	37.51	1.3486	HLA-B*08:01

**Table 2:** The final set of helper T lymphocyte (HTL) epitopes selected for multi-epitope vaccine construction.

Protein	Peptide	Core peptide	Antigenicity	Allele
S	^511^VVLSFELLHAPATVC^525^	FELLHAPAT	0.8618	HLA-DRB1*01:01
S	^166^CTFEYVSQPFLMDLE^180^	EYVSQPFLM	0.57	HLA-DPA1*03:01/DPB1*04:02; HLA- DPA1*02:01/DPB1*01:01
S	^750^SNLLLQYGSFCTQLN^764^	LLQYGSFCT	0.8305	HLA-DRB1*15:01
S	^168^FEYVSQPFLMDLEGK^182^	EYVSQPFLM	0.8278	HLA-DPA1*03:01/DPB1*04:02
S	^751^NLLLQYGSFCTQLNR^765^	LLQYGSFCT	0.8668	HLA-DRB1*15:01
S	^142^GVYYHKNNKSWMESE^156^	YHKNNKSWM	0.4684	HLA-DRB3*02:02
S	^141^LGVYYHKNNKSWMES^155^	YHKNNKSWM	0.4937	HLA-DRB3*02:02
S	^1210^IKWPWYIWLGFIAGL^1224^	YIWLGFIAG	0.9153	HLA-DPA1*01:03/DPB1*02:01
S	^140^FLGVYYHKNNKSWME^154^	YHKNNKSWM	0.4793	HLA-DRB3*02:02
S	^346^RFASVYAWNRKRISN^360^	FASVYAWNR	0.4243	HLA-DRB5*01:01
S	^55^FLPFFSNVTWFHAIH^69^	FSNVTWFHA	0.4883	HLA-DPA1*02:01/DPB1*01:01
S	^166^CTFEYVSQPFLMDLE^180^	YVSQPFLMD	0.57	HLA-DPA1*01:03/DPB1*04:01; HLA-DPA1*01:03/DPB1*02:01
N	^83^QIGYYRRATRRIRGG^97^	YRRATRRIR	0.4614	HLA-DRB1*11:01; HLA-DRB5*01:01
N	^303^QIAQFAPSASAFFGM^317^	FAPSASAFF	0.4032	HLA-DRB1*09:01

### Multi-epitope vaccine polypeptide construction

**Figure 2:**
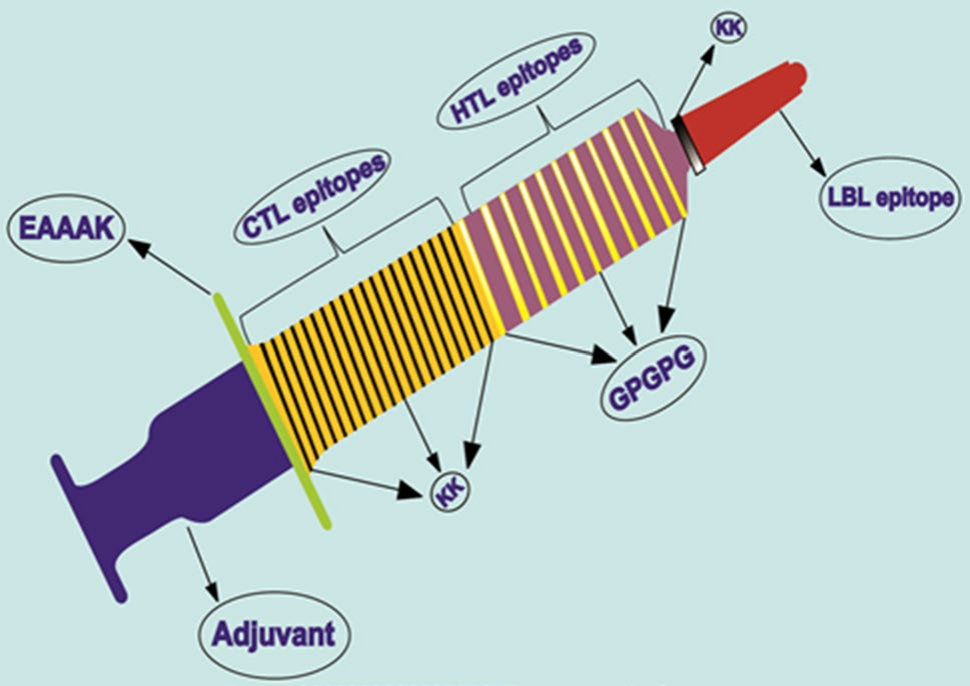
Schematic profile of the multi-epitope subunit vaccine construct of length 694 residues. An adjuvant was added at the N-terminal tail of the vaccine using EAAAK linker, followed by 34 CTL, 12 HTL and 1 LBL epitopes fused by KK and GPGPG linkers.

The total of 34 CTL, and 12 HTL epitopic peptides were fused to each other by KK, and GPGPG linkers, respectively, followed by adjoining a single LBL epitope using the KK linker, to create the multi-epitope peptide-based vaccine construct^12–14^. Furthermore, to boost the immunogenicity of the multi-epitope vaccine, β-defensin (GIINTLQKYYCRVRGGRCAVLSCLPKEEQIGKCSTRGRKCCRRKK) was added as an adjuvant to the amino terminus of the polypeptide using an EAAAK linker to the first CTL epitope. The primary structure of the multi-epitope subunit vaccine construct included the total of 694 amino acids (Fig. 2 and Supplementary Fig. S1 online).

### Immunogenicity, allergenicity and physiochemical properties of the vaccine candidate

Assessment of immunogenic, allergenic and solubility showed the multi-epitope vaccine candidate was of a suitable design (Table 3). The calculated molecular weight of the final vaccine polypeptide (78.35 kDa) indicated its good antigenic nature^13,15^, and the pI value of 10.38 showed the basic nature of the final vaccine candidate. The instability index of the final polypeptide (36.73) indicated its high stability. The Grand average of hydropathicity value was − 0.261, showing that the final structure is slightly hydrophilic in nature, which can lead to better connection with other proteins. Moreover, the aliphatic index of the peptide was equal to 81.44, which implied the final peptide had high thermo-stability (Table 3). Estimated half-life was 30 h in mammalian reticulocytes in vitro, and > 20 h in yeast, and > 10 h in *E. coli *in vivo.

**Table 3:** Antigenic, allergenic and physiochemical assessments of the primary sequence of final vaccine protein.

Features	Assessment
Number of amino acids	694
Molecular weight	78,351.68 Dalton
Theoretical pI	10.37
No. of negatively charged residues (Asp + Glu)	25
No. of positively charged residues (Arg + Lys)	137
Extinction coefficient (at 280 nm in H_2_O)	120,305 M^−1^ cm^−1^
Instability index	36.73
Aliphatic index	81.44
Grand average of hydropathicity (GRAVY)	− 0.261
Antigenicity	0.7342 (VaxiJen v.2.0)
Allergenicity	Probable non-allergen (AllergenFP v.1.0) Probable non-allergen (AllerTOP v.2.0)
Solubility	0.914870 (SOLpro)

### 3D structure of the vaccine polypeptide

The sequence of the multi-epitope vaccine polypeptide was submitted to appropriate tools, and a refined model of its 3D structure was obtained (Supplementary Fig. S1 online). The output from ProSA-web validation tools showed a Z-score of -4.33, indicating the good quality of the final vaccine polypeptide structure (Supplementary Fig. S1 online). In addition, RAMPAGE analysis indicated that 92.3% amino acids of the final structure were in the favoured area, 6.1% were in the allowed area, and 1.6% were in the disallowed area of Ramachandran plot, reflecting a high structural quality of the constructed vaccine model (Supplementary Fig. S1 online).

### Conformational and linear B-cell epitopes in the vaccine polypeptide structure

**Figure 3:**
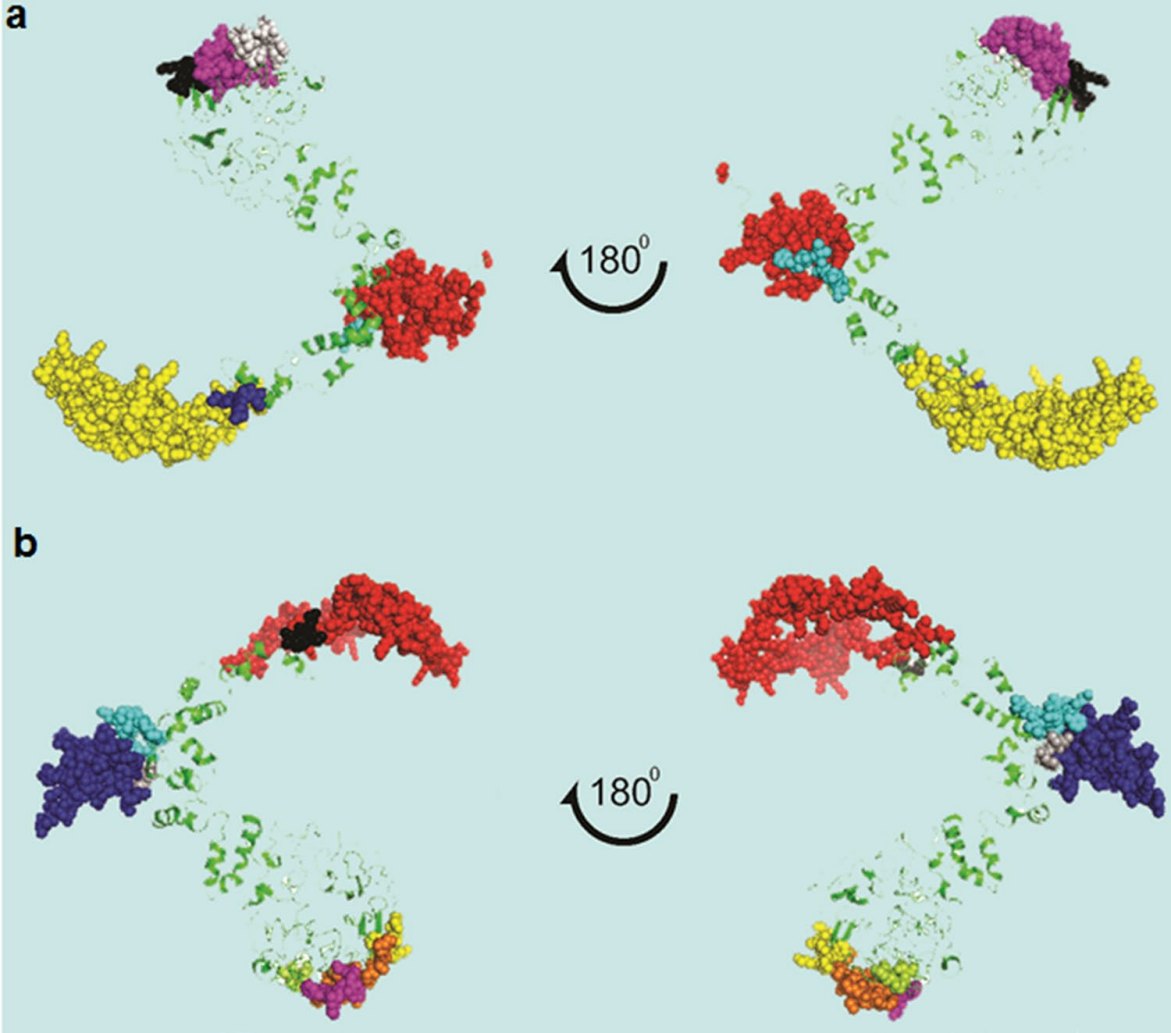
B lymphocyte epitopes present in the designed multi-epitope vaccine. (**a**) Conformational B cell epitopes shown by spheres, (**b**) Linear B cell epitopes shown by spheres.

The total of 7 conformational epitopes with scores of 0.702 to 0.882, and 9 linear epitopes with scores of 0.7 to 0.878 were selected as the final B-cell epitopes (Fig. 3, Supplementary Table S7 online). PI value (the score given by ElliPro) of 0.882 shows that 88.2% residues are locating in the predicted ellipsoid area of the epitope and this epitope features the highest solvent accessibility.

### Binding of the vaccine structure to TLR-3

The best docked complex of the immune receptor TLR-3 with the vaccine model was identified from among various server outputs by comparing the binding free energy. Analysis of the residues contributing at the protein–protein interface showed that the C-terminal domain of the polypeptide is involved in the interaction, where residues from vaccine polypeptide form polar or non-polar contacts with three domains on the TLR-3 structure (Supplementary Fig. S2 online). The docked complex was further applied for running molecular dynamic (MD) simulation investigations.

### MD relaxation and analysis of the receptor-vaccine complex

The MD simulations of the docked multi-epitope-based subunit vaccine with TLR-3 as the receptor were carried out to achieve information about the conformational changes of TLR-3-vaccine polypeptide complex. Such studies were essential for several vital facets: (1) to comprehend whether the designed vaccine was stable at the bound pocket; (2) to verify that the induced conformational mobility of both TLR-3 receptor and the multi-epitope vaccine structure did not exert undesired impact on the conformation of the docked proteins; and (3) to corroborate that the epitopes of the multi-epitope vaccine were potential for efficiently being recognized by the human immune system, causing strong immune response.

**Figure 4:**
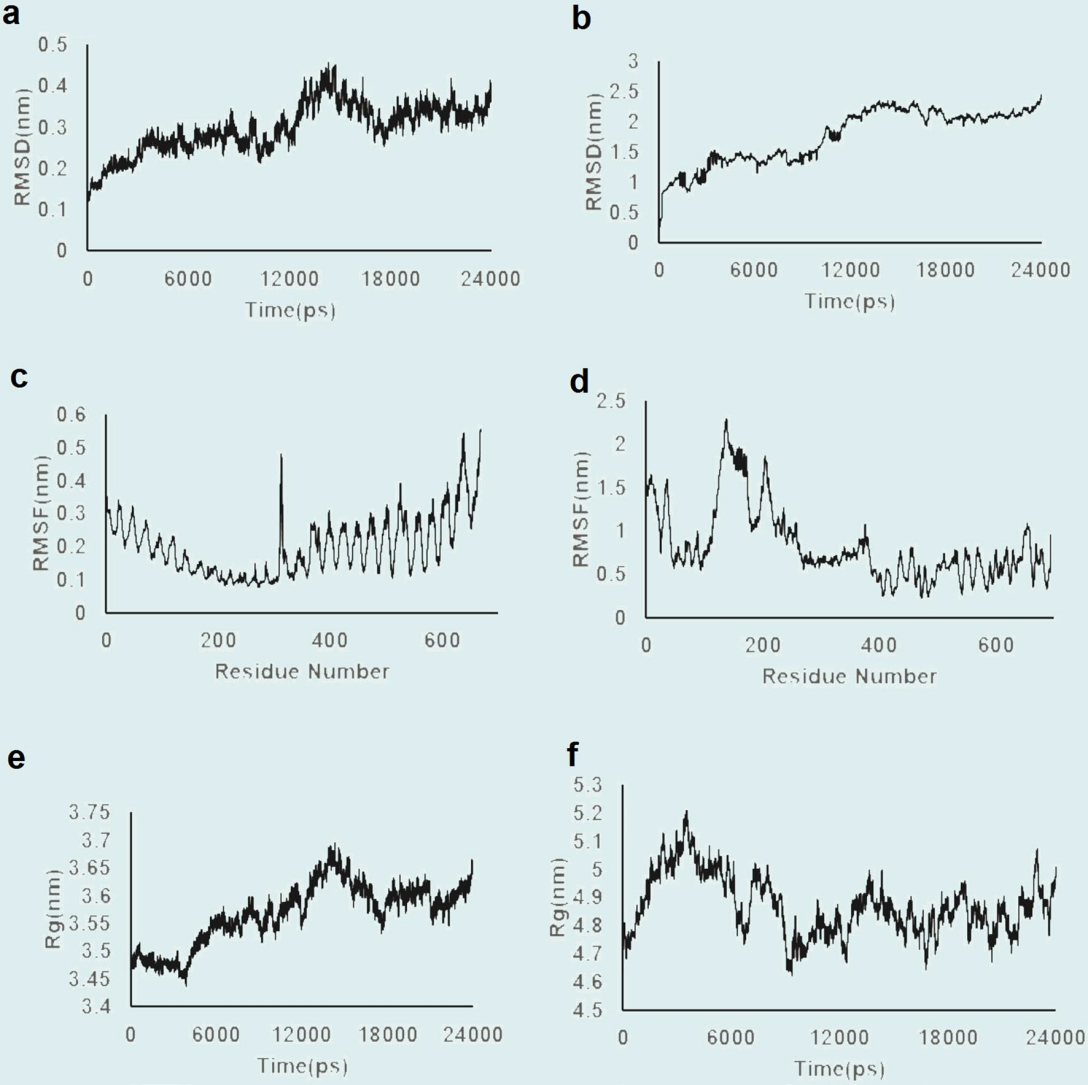
Illustration of the molecular dynamic equilibration for simulation outputs. (**a**) Root mean squared deviations (RMSDs) of Cα for Toll-like receptor-3 (TLR-3), and (**b**) for the multi-epitope vaccine polypeptide; (**c**) Root mean squared fluctuations (RMSFs) of Cα atoms for TLR-3, and (**d**) for the multi-epitope vaccine polypeptide; (**e**) Radius of gyration for TLR-3, and (**f**) for the multi-epitope vaccine polypeptide.

Three statistical factors were assessed based on 24,000 ps of simulation trajectory (Fig. 4). The root mean squared deviation (RMSD) values of TLR-3 and multi-epitope vaccine in the complex reflected the high conformational stability of the docked molecules. An average RMSD of 0.29 nm with maximum of 0.44 nm realized at 14,000 ps was noted for the TLR-3 molecule (Fig. 4A). The RMSD value for the multi-epitope vaccine model (Fig. 4B) showed that it mostly remained stable during simulation time, with a plateau at about 10,500 ps. The observed trends can be attributed to the moving multi-epitope vaccine at TLR-3 binding pocket in an effort to obtain a suitable and stable docked conformation.

Region-wise structural fluctuations of the TLR-3-vaccine polypeptide complex were studied by calculating the root mean squared fluctuation (RMSF) parameter (Fig. 4C,D). The mean RMSF calculated for TLR-3 and the multi-epitope vaccine were 0.2 nm and 0.83 nm, respectively, which were overall in favour of protein residues’ local stability. Along the vaccine sequence, residues located at the loop regions (such as I137, T138, W204, N376 and R654) had high fluctuation (Fig. 4D). The flexibility of the loop regions is essential for proper holding of the vaccine at the binding pocket.

The compactness of the complex structure was estimated by calculating the radius of gyration (Rg) of TLR-3 and multi-epitope peptide molecules. The related graphs (Fig. 4E,F) showed that during the simulation time, TLR-3 and multi-epitope vaccine molecules had mean Rg values of about 3.56 nm and 4.87 nm, respectively. Rg fluctuations is an indication of movements and conformational changes in flexible regions of the multi-epitope vaccine peptide in the TLR-3 binding pocket. This dynamics seems essential to suitably identify the vaccine and incorporating it in the binding pocket.

### Free energy of the binding between vaccine polypeptide and TLR-3

To figure out the strength of the contact between multi-epitope vaccine and TLR-3 structures, the binding free energy between the two molecules was calculated using MMPBSA approach. According to Table 4, the nonpolar element (− 136.92 kcal/mol) was an important energy term in the binding free energy of the complex. Our findings clarified that the favourable electrostatic energy (E_ele_ = − 241.5 kcal/mol) was covered up by the huge polar energy component (ΔG_GB_ = 240.3 kcal/mol) in the binding process of the multi-epitope vaccine polypeptide. Therefore, the nonpolar energy was known as the main driving force in the vaccine binding to TLR-3, and this hydrophobic contribution leads to a thermodynamically favourable interaction (∆G_binding_ = − 138.11 kcal/mol). To further clarify the binding mode, the calculated energy was broken down into residue-residue pairs through the binding free energy decomposition analysis. According to the data, there were several residues of vaccine polypeptide with less than − 1.5 kcal/mol free energy of contribution in the binding mechanism. The binding pose of vaccine with the key residues are illustrated in Fig. S3.

**Table 4:** Binding free energy calculation for the multi-epitope vaccine candidate-TLR-3 complex.

	Energy values (kcal/mol)
∆E_ele_^a^	− 241.5 ± 14.81
∆E_vdW_^b^	− 118.81 ± 9.0
∆G_GB_^c^	240.3 ± 16.71
∆G_SA_^d^	− 18.11 ± 1.1
∆E_polar_^e^	− 1.2 ± 0.4
∆E_non-polar_^f^	− 136.92 ± 4.07
∆G_binding_	− 138.11 ± 9.46

^a^Electrostatic contribution.

^b^van der Waals contribution.

^c^Polar contribution of the solvation effect.

^d^Non-polar contribution of solvation effect.

^e^∆E_polar_ = ∆E_ele_ + ∆G_GB_.

^f^∆E_non-polar_ = ∆E_vdW_ + ∆G_SA_.

## Discussion

Classic live or attenuated vaccines have a long history of success, however their deployment is associated with issues of biosafety such as autoimmune or strong allergic responses, plus difficulties with their synthesis and manufacture. These drawbacks might be addressed by developing fully synthetic peptide-based vaccines^16^. Research on COVID-19 prevention through this approach was begun immediately after release of relevant basic data such as its full genomic sequence. Specific epitope regions in SARS-CoV-2 with high homology to SARS-CoV, the best-characterized coronavirus in terms of epitope responses, were identified^17–19^. Antigenic properties of spike glycoprotein were more focused by theory and experimental researchers^20–22^. Applying this basic knowledge, a number of peptide-based designs have been presented to date, which encompass those integrating epitopes from a single or few viral protein(s)^23–27^ to those considering the whole proteome of the virus^28^. The present research contributes to current understanding in this field by offering an alternative approach for developing a potential COVID-19 vaccine, where the entire set of structural proteins were searched for most efficacious epitopic segments.

One rationale for the choice of the whole set of structural proteins of SARS-CoV-2 for epitope identification in this study, was the ample evidence on immunity-related advantages of each of the S, E, M and N proteins of SARS-CoV or SARS-CoV-2^17,29–32^. In addition, the choice of structural proteins as the source of epitopes implies a highly-conserved epitope set, as structural proteins are subject to less sequence or structural evolution^18^. We found that our proposed epitopes demonstrate overlap with similar studies which have extracted the epitopes from individual structural proteins from different sequence database entries; the similarity of findings and the conservation analyses by those research groups may also indicate the high epitope conservancy among geographical strains^26,27^. Aside from these advantages, our selection of structural epitopes aimed to cope with potential vaccine side effects, particularly the antibody-dependent enhancement of infectivity (ADEI). ADEI is a great challenge of subunit vaccines, referring to the reduced specificity in response elicitation because of the numerous conformations adopted by a peptide vaccine^10^. Strategies to mitigate this concern include: 1- immunofocusing by considering several most antigenic epitopes^33^; 2- inclusion of structural proteins: structurally flexible coronaviral epitopes may be of limited value for in vivo immunotargeting and require to be replaced by conserved epitopes with low structural plasticity^6^. Structural proteins are characterized by highly conserved sequences, thus the linear epitopes remain highly stable in these proteins and the conformational epitopes preserve their structural patterns during various steps of the virus cycle. Our choice to include the full set of SARS-CoV-2 structural proteins has thus combined the advantages of augmented immunogenicity and geographical conservancy with precautions of both strategies proposed for reducing ADEI.

Integrating multiple immunodominant sites of viral pathogens in the vaccine structure helps augment the antigenic effect. Reports revealed that multiple antigenic peptides induce stronger B and T cells immune responses than un-conjugated peptide epitopes^34–36^. Thus, consecutive sequences of HTL and CTL epitopic peptides were fused to each other using accepted linkers. GPGPG has been introduced as a spacer between adjacent epitopes, because of several positive contributions to the immune-modulatory function of polypeptide vaccines, including reduction of junctional epitopes, increased proteasome processing, and augmented immunogenicity^37^. The KK linker is the target sequence of cathepsin B, a lyzosomal protease of high importance for antigen processing. Connecting two peptide antigens together using the lysine linker helps avoid induction of antibodies to the amino acid sequence that is generated by joining of two peptides and most antibodies would be reactive to each peptide^38^.

Epitope-based peptide vaccines induce relatively weak immune response, when used alone^39^. The immunoreactivity could be improved by appending proper adjuvants. The vaccine construct in this study was prepared by fusing the epitopic peptides to β-defensin as an immunogenic adjuvant. β-Defensin has previously been reported as a potent adjuvant when conjugated with MERS-CoV antigens. Vaccines containing defensins as adjuvants have been shown, both in vivo and in vitro, to activate the primary innate antiviral immune response and mediate other immunomodulatory activities against a number of viruses, including coronaviruses^40,41^. Use of appropriate adjuvants has also been shown to help induce durable IFN-γ responses^6^.

In this study, the binding of the built vaccine model with the immune receptor TLR-3 was assessed by performing molecular docking, MD simulation and free energy calculations. Triggering TLR-3 may help induce the TLR signalling networks which activate the immune pathways evolved specifically against viral pathogens. In addition, this TLR choice was a consensus from vaccine design research works on viral/retroviral pathogens, specifically coronavirus species^12,14,25,26,42,43^. While we note that other Toll-like immune receptors such as TLR-2, -4, -5, -7 and -8 may be stimulated by the COVID-19 virus, they were not considered here as their exact roles are not established, with some possibly functioning even to the advantage of the coronavirus^44,45^.

## Conclusion

In the ongoing urgent situation brought about by SARS-CoV-2, it is hard to fast counteract the circulating disease through preventative or therapeutic measures. The multi-epitope-based subunit vaccine design obtained through an immunoinformatic pipeline in this study could be promising, as it incorporates a priori bioinformatics predictions and up-to-date immunological knowledge. Despite the current highly active research community as well as the efforts of the industry section in the road to find an optimal immunogenic formulation, the extreme diversity in available design choices, with no a priori image of their effectiveness, appears as a fundamental obstacle in this route. To overcome this issue, a rapid mass design and screening strategy incorporating the same immunoinformatic approach is recommended to be developed by the research community, with the hope to find one formulation with the highest immunogenicity and biosafety.

## Methodology

### SARS-CoV-2 structural protein sequences

The amino acid sequences of SARS-CoV-2 structural proteins, including the spike glycoprotein (S), envelope protein (E), membrane protein (M), and nucleocapsid phosphoprotein (N), were retrieved using the NCBI reference genome of the virus (accession number NC_045512.2).

### Identifying cytotoxic T lymphocyte (CTL) epitopes

Predicting peptides that are capable of inducing CTL responses is a crucial step in the design of epitope-based vaccine. The MHC-I Binding tool of Immune Epitope Database and Analysis Resource (IEDB; http://tools.iedb.org/mhci) was used to predict the CD8^+^ T-cell epitopes borne in S, E, M and N proteins^12,46,47^. In this step, the ANN 4.0 method was set as the prediction method. The human was selected as the source species. The maximum IC_50_ value was set to 50 nM, and percentile rank < 1 was considered as threshold since lower score indicates high affinity^48,49^.

### Identifying helper T lymphocyte (HTL) epitopes

IEDB (http://www.iedb.org) was used to predict MHC-II binding of 15-mer epitopes for viral structural proteins against human HLAs such as HLA-DRB1*15:01, HLA-DRB4*01:01, HLA-DRB3*01:01, HLA-DRB5*01:01, HLA-DRB1*03:01, HLA-DRB3*02:02 and HLA-DRB1*07:01 (Supplementary Table S8 online), using NN-align 2.3 method^13,14,50,51^. The maximum IC_50_ value of 50 nM, 500 nM and 5000 nM indicate high, intermediate and low affinity of epitopes, respectively^52^. The 15-mer epitopes with IC_50_ values < 50 nM were considered to be included in the vaccine polypeptide^52–54^.

### B-cell epitopes prediction

The ABCpreds server was used to predict 16-mer linear B-lymphocyte (LBL) epitopes, using a threshold of 0.51^55^. In addition, the ElliPro tool of IEDB was utilized to predict conformational and linear B-cell epitopes of the vaccine polypeptide.

### Assessment of identified epitopes for antigenicity, allergenicity, and toxicity

The antigenic potential of each of the T and B cells epitopes was predicted by VaxiJen v2.0 applying a threshold of 0.4^56^. The predicted T and B cells epitopes were then further evaluated in terms of toxicity and allergenicity, with ToxinPred and AllergenFP v1.0 servers, respectively^57,58^. Then, the ability of each of the HTL and B cell epitopes (CD4^+^) to induce the secretion of cytokines, such as interferon-gamma (IFN-γ), interleukin-4 (IL-4) and interleukin-10 (IL-10), to overcome the inflammatory response and prevent tissue damage was predicted by IFNepitope, IL4pred and IL10pred server tools, respectively^59–61^. The IL4pred and IL10pred operations were carried out based on SVM method and hybrid method, respectively, with other parameters kept as default^12,13^.

### Designing the multi-epitope vaccine polypeptide construct

To develop a multi-epitope vaccine construct, we selected those predicted epitopes which demonstrated high antigenic potential, were not identified as allergic or toxic, and had good solubility when highly expressed. To construct the vaccine polypeptide, the selected CTL, HTL and LBL epitopes were fused together using appropriate linkers^62,63^. In addition, a 45-amino acid sequence prepared from β-defensin-2 protein was added to the N terminus of the vaccine sequence using the EAAAK linker, to increase the immunogenic capacity of the multi-epitope vaccine^12,52,63^.

### Immunogenic, allergenic and physiochemical evaluation of vaccine construct

The antigenicity of the multi-epitope vaccine polypeptide was predicted utilizing the VaxiJen v2.0 tool, with the threshold value of 0.4^56^. The allergenicity of the vaccine was analysed using AllerTOP v.2.0 and AllergenFP v.1.0 servers^57,64^. The ProtParam server was employed to evaluate the physical chemistry properties of the vaccine construct, such as amino acid composition, molecular weight, theoretical isoelectric point (pI), grand average of hydropathicity (GRAVY), aliphatic and instability index, and in vitro and in vivo half-life^65^.

### Vaccine polypeptide structure modelling, refinement and validation

The SOPMA server was applied for analysing the secondary structural properties of the multi-epitope vaccine polypeptide^66^. The GalaxyWEB server was employed for modelling and refinement of the 3D structure model^67^. The server relaxes the model structure using repacking and molecular dynamics (MD) simulation. Next, RAMPAGE server and ProSA-web tools were used to validate the refined 3D model^68,69^. All of these tools give us the overall quality of the 3D structure of the peptide vaccine.

### Molecular docking of the vaccine polypeptide to TLR-3

The binding of antigenic agents with the target immune cell protein is crucial for the creation of a suitable immune system response. For analysing the binding pattern of the multi-epitope vaccine polypeptide with TLR-3 (PDB ID: 2A0Z)^70^, molecular docking analysis was performed by Hdock, Zdock, Cluspro and Hawkdock^71–74^. Among the molecular species docked by each of these applications, the best outputs were extracted, followed by the four docked molecules uploaded into the Hawkdock program, and the free energy of binding of each complex calculated. Based on this screening, it was determined that the model selected from the Cluspro output had the best binding free energy. Therefore, this molecular species was chosen as the primary structure for initiating the MD simulation.

### MD simulation of the vaccine-receptor complex

To determine the structural stability and to study the molecular details of the interactions between TLR-3 and the multi-epitope vaccine polypeptide in the docked conformation, MD simulation was performed. Briefly, the system including vaccine polypeptide-TLR-3 was simulated by the Gromacs-2020 package applying OPLS-AA force field^75^. The complex system was solvated using TIP3P water model. Then, the genion module was utilized to neutralize the whole system. Next, the conjugate gradient algorithm was applied to minimize the energy of the system. In an NVT ensemble, the temperature of the system gradually increased from 0 to 310 K during 400 ps. Subsequently, in an NPT ensemble, 500 ps simulation was carried out at the pressure of 1 atm and the temperature of 310 K. Production simulation for 24,000 ps was then implemented. The particle-mesh Ewald (PME) and the LINCS algorithms were applied to assess all electrostatic connections and to restrain all bond lengths in the protein, respectively. Moreover, periodic boundary condition was utilized during the simulation. The final coordinates obtained for the complex system were analysed with classic MD analyses, plus the MMPBSA method for calculating the free energy of intermolecular interactions^76^. The results were visualized by Pymol (Schrodinger L.L.C.).

## Supplementary information

Supplementary Information.

## Data Availability

No datasets were generated or analysed during the current study. Data such as epitope sequences, which were analysed during this study, are included in this published article and its Supplementary Information file.

## References

[1] Cui J, Li F, Shi ZL (2019). Origin and evolution of pathogenic coronaviruses. Nat. Rev. Microbiol..

[2] World Health Organization. *Coronavirus disease 2019 (COVID-19), Situation reports, Weekly Epidemiological Update, 21 September 2020*. www.who.int/docs/default-source/coronaviruse/situation-reports/20200921-weekly-epi-update-6.pdf?sfvrsn=d9cf9496_6 (2020).

[3] Tseng CT (2012). Immunization with SARS coronavirus vaccines leads to pulmonary immunopathology on challenge with the SARS virus. PLoS ONE.

[4] Muthumani K (2015). A synthetic consensus anti-spike protein DNA vaccine induces protective immunity against Middle East respiratory syndrome coronavirus in nonhuman primates. Sci. Transl. Med..

[5] Wang L (2015). Evaluation of candidate vaccine approaches for MERS-CoV. Nat. Commun..

[6] Enjuanes L (2016). Molecular basis of coronavirus virulence and vaccine development. Adv. Virus Res..

[7] Wang J (2012). The adjuvanticity of an *O. volvulus*-derived rOv-ASP-1 protein in mice using sequential vaccinations and in non-human primates. PLoS ONE.

[8] Zhang N (2016). Identification of an ideal adjuvant for receptor-binding domain-based subunit vaccines against Middle East respiratory syndrome coronavirus. Cell. Mol. Immunol..

[9] Papaneri AB (2015). Middle East respiratory syndrome: obstacles and prospects for vaccine development. Expert Rev. Vaccines.

[10] Graham BS, Gilman MSA, McLellan JS (2019). Structure-based vaccine antigen design. Annu. Rev. Med..

[11] Khan MK (2014). In silico predicted mycobacterial epitope elicits in vitro T-cell responses. Mol. Immunol..

[12] Abdulla F, Adhikari UK, Uddin MK (2019). Exploring T & B-cell epitopes and designing multi-epitope subunit vaccine targeting integration step of HIV-1 lifecycle using immunoinformatics approach. Microb Pathog.

[13] Khatoon N, Pandey RK, Prajapati VK (2017). Exploring Leishmania secretory proteins to design B and T cell multi-epitope subunit vaccine using immunoinformatics approach. Sci. Rep..

[14] Narula A, Pandey RK, Khatoon N, Mishra A, Prajapati VK (2018). Excavating chikungunya genome to design B and T cell multi-epitope subunit vaccine using comprehensive immunoinformatics approach to control chikungunya infection. Infect. Genet. Evol..

[15] Berzofsky, J. A. & Berkower, I. in *Fundamental Immunology* Vol. 631–684 (ed W.E. Paul) (Raven Press, 1993).

[16] Skwarczynski M, Toth I (2016). Peptide-based synthetic vaccines. Chem. Sci..

[17] Grifoni A (2020). A sequence homology and bioinformatic approach can predict candidate targets for immune responses to SARS-CoV-2. Cell Host Microbe.

[18] Kumar S, Maurya VK, Prasad AK, Bhatt MLB, Saxena SK (2020). Structural, glycosylation and antigenic variation between 2019 novel coronavirus (2019-nCoV) and SARS coronavirus (SARS-CoV). Virusdisease.

[19] Ahmed SF, Quadeer AA, McKay MR (2020). Preliminary identification of potential vaccine targets for the covid-19 coronavirus (SARS-CoV-2) based on SARS-CoV immunological studies. Viruses.

[20] Baruah V, Bose S (2020). Immunoinformatics-aided identification of T cell and B cell epitopes in the surface glycoprotein of 2019-nCoV. J. Med. Virol..

[21] Lucchese G (2020). Epitopes for a 2019-nCoV vaccine. Cell. Mol. Immunol..

[22] Walls AC (2020). Structure, function, and antigenicity of the SARS-CoV-2 spike glycoprotein. Cell.

[23] Bhattacharya M (2020). Development of epitope-based peptide vaccine against novel coronavirus 2019 (SARS-COV-2): Immunoinformatics approach. J. Med. Virol..

[24] Kar T (2020). A candidate multi-epitope vaccine against SARS-CoV-2. Sci. Rep..

[25] Rahman MS (2020). Epitope-based chimeric peptide vaccine design against S, M and E proteins of SARS-CoV-2, the etiologic agent of COVID-19 pandemic: an in silico approach. PeerJ.

[26] Kalita P, Padhi AK, Zhang KYJ, Tripathi T (2020). Design of a peptide-based subunit vaccine against novel coronavirus SARS-CoV-2. Microb. Pathog..

[27] Lizbeth RG, Jazmin GM, Jose CB, Marlet MA (2020). Immunoinformatics study to search epitopes of spike glycoprotein from SARS-CoV-2 as potential vaccine. J. Biomol. Struct. Dyn..

[28] Crooke SN, Ovsyannikova IG, Kennedy RB, Poland GA (2020). Immunoinformatic identification of B cell and T cell epitopes in the SARS-CoV-2 proteome. Sci. Rep..

[29] Liu J (2010). The membrane protein of severe acute respiratory syndrome coronavirus acts as a dominant immunogen revealed by a clustering region of novel functionally and structurally defined cytotoxic T-lymphocyte epitopes. J. Infect. Dis..

[30] Ng OW (2016). Memory T cell responses targeting the SARS coronavirus persist up to 11 years post-infection. Vaccine.

[31] Peng H (2006). Human memory T cell responses to SARS-CoV E protein. Microb. Infect..

[32] Surjit M, Lal SK (2008). The SARS-CoV nucleocapsid protein: a protein with multifarious activities. Infect. Genet. Evol..

[33] Ma C (2014). Searching for an ideal vaccine candidate among different MERS coronavirus receptor-binding fragments: the importance of immunofocusing in subunit vaccine design. Vaccine.

[34] Tam JP (1988). Synthetic peptide vaccine design: synthesis and properties of a high-density multiple antigenic peptide system. Proc. Natl. Acad. Sci..

[35] Li W, Joshi MD, Singhania S, Ramsey KH, Murthy AK (2014). Peptide vaccine: progress and challenges. Vaccines.

[36] Reche PA, Fernandez-Caldas E, Flower DR, Fridkis-Hareli M, Hoshino Y (2014). Peptide-based immunotherapeutics and vaccines. J. Immunol. Res..

[37] Livingston B (2002). A rational strategy to design multiepitope immunogens based on multiple Th lymphocyte epitopes. J. Immunol..

[38] Yano A (2005). An ingenious design for peptide vaccines. Vaccine.

[39] Tahir Ul Qamar M (2019). Epitope-based peptide vaccine design and target site depiction against Middle East Respiratory Syndrome Coronavirus: an immune-informatics study. J. Transl. Med..

[40] Kim J, Yang YL, Jang SH, Jang YS (2018). Human beta-defensin 2 plays a regulatory role in innate antiviral immunity and is capable of potentiating the induction of antigen-specific immunity. Virol. J..

[41] Park MS, Kim JI, Lee I, Park S, Bae JY (2018). Towards the application of human defensins as antivirals. Biomol. Ther..

[42] Srivastava S, Kamthania M, Singh S, Saxena AK, Sharma N (2018). Structural basis of development of multi-epitope vaccine against Middle East respiratory syndrome using in silico approach. Infect. Drug Resist..

[43] Ikram A (2018). Exploring NS3/4A, NS5A and NS5B proteins to design conserved subunit multi-epitope vaccine against HCV utilizing immunoinformatics approaches. Sci. Rep..

[44] Olejnik J, Hume AJ, Muhlberger E (2018). Toll-like receptor 4 in acute viral infection: too much of a good thing. PLoS Pathog..

[45] Imai Y (2008). Identification of oxidative stress and Toll-like receptor 4 signaling as a key pathway of acute lung injury. Cell.

[46] Larsen MV (2007). Large-scale validation of methods for cytotoxic T-lymphocyte epitope prediction. BMC Bioinform..

[47] Yadav G, Rao R, Raj U, Varadwaj PK (2017). Computational modeling and analysis of prominent T-cell epitopes for assisting in designing vaccine of ZIKA virus. J. Appl. Pharm. Sci..

[48] Andreatta M, Nielsen M (2016). Gapped sequence alignment using artificial neural networks: application to the MHC class I system. Bioinformatics.

[49] Nielsen M, Lund O (2009). NN-align. An artificial neural network-based alignment algorithm for MHC class II peptide binding prediction. BMC Bioinform..

[50] Greenbaum J (2011). Functional classification of class II human leukocyte antigen (HLA) molecules reveals seven different supertypes and a surprising degree of repertoire sharing across supertypes. Immunogenetics.

[51] Weiskopf D (2013). Comprehensive analysis of dengue virus-specific responses supports an HLA-linked protective role for CD8+ T cells. Proc. Natl. Acad. Sci. USA..

[52] Duvvuri VR (2014). Preexisting CD4+ T-cell immunity in human population to avian influenza H7N9 virus: whole proteome-wide immunoinformatics analyses. PLoS ONE.

[53] Shey RA (2019). In-silico design of a multi-epitope vaccine candidate against onchocerciasis and related filarial diseases. Sci. Rep..

[54] Zhou WY (2009). Therapeutic efficacy of a multi-epitope vaccine against *Helicobacter pylori* infection in BALB/c mice model. Vaccine.

[55] Saha S, Raghava GP (2006). Prediction of continuous B-cell epitopes in an antigen using recurrent neural network. Proteins.

[56] Doytchinova IA, Flower DR (2007). VaxiJen: a server for prediction of protective antigens, tumour antigens and subunit vaccines. BMC Bioinform..

[57] Dimitrov I, Naneva L, Doytchinova I, Bangov I (2014). AllergenFP: allergenicity prediction by descriptor fingerprints. Bioinformatics.

[58] Gupta S (2013). In silico approach for predicting toxicity of peptides and proteins. PLoS ONE.

[59] Dhanda SK, Gupta S, Vir P, Raghava GP (2013). Prediction of IL4 inducing peptides. Clin. Dev. Immunol..

[60] Dhanda SK, Vir P, Raghava GP (2013). Designing of interferon-gamma inducing MHC class-II binders. Biol Direct.

[61] Nagpal G (2017). Computer-aided designing of immunosuppressive peptides based on IL-10 inducing potential. Sci. Rep..

[62] Chen X, Zaro JL, Shen WC (2013). Fusion protein linkers: property, design and functionality. Adv. Drug Deliv. Rev..

[63] Mohan T, Sharma C, Bhat AA, Rao DN (2013). Modulation of HIV peptide antigen specific cellular immune response by synthetic alpha- and beta-defensin peptides. Vaccine.

[64] Dimitrov I, Bangov I, Flower DR, Doytchinova I (2014). AllerTOP vol 2–a server for in silico prediction of allergens. J. Mol. Model.

[65] Gasteiger, E. *et al.* in *The Proteomics Protocols Handbook Springer Protocols Handbooks* (ed J.M. Walker) 571–607 (Humana Press, 2005).

[66] Geourjon C, Deleage G (1995). SOPMA: significant improvements in protein secondary structure prediction by consensus prediction from multiple alignments. CABIOS.

[67] Ko J, Park H, Heo L, Seok C (2012). GalaxyWEB server for protein structure prediction and refinement. Nucleic Acids Res..

[68] Wang W (2016). Data set for phylogenetic tree and RAMPAGE Ramachandran plot analysis of SODs in *Gossypium raimondii* and *G. arboreum*. Data Brief.

[69] Wiederstein M, Sippl MJ (2007). ProSA-web: interactive web service for the recognition of errors in three-dimensional structures of proteins. Nucleic Acids Res..

[70] Bell JK (2005). The molecular structure of the Toll-like receptor 3 ligand-binding domain. Proc. Natl. Acad. Sci. USA..

[71] Kozakov D (2017). The ClusPro web server for protein-protein docking. Nat. Protoc..

[72] Pierce BG, Hourai Y, Weng Z (2011). Accelerating protein docking in ZDOCK using an advanced 3D convolution library. PLoS ONE.

[73] Weng G (2019). HawkDock: a web server to predict and analyze the protein-protein complex based on computational docking and MM/GBSA. Nucleic Acids Res..

[74] Yan Y, Zhang D, Zhou P, Li B, Huang SY (2017). HDOCK: a web server for protein-protein and protein-DNA/RNA docking based on a hybrid strategy. Nucleic Acids Res..

[75] Van Der Spoel D (2005). GROMACS: fast, flexible, and free. J. Comput. Chem..

[76] Kumari R, Kumar R, Lynn A (2014). g_mmpbsa–a GROMACS tool for high-throughput MM-PBSA calculations. J. Chem. Inf. Model..

