# Supplementary material for: Immunoinformatic design of a COVID-19 subunit vaccine using entire structural immunogenic epitopes of SARS-CoV-2
Source: Sci Rep. 2020 Nov 30;10:20864. doi: 10.1038/s41598-020-77547-4 (PMC7704662; doi:10.1038/s41598-020-77547-4)
Supplement: Supplementary file 1 — Supplementary Information. [file 41598_2020_77547_MOESM1_ESM.pdf]

Immunoinformatic design of a COVID-19 subunit vaccine using entire structural immunogenic epitopes of SARS-CoV-2

Esmaeil Behmard,[a,b] Bijan Soleymani,[a] Ali Najafi,[c] Ebrahim Barzegari [a,*]

[a] Medical Biology Research Center, Health Technology Institute, Kermanshah University of Medical Sciences, Kermanshah, Iran

[b] Pharmaceutical Sciences Research Center, Shiraz University of Medical Sciences, Shiraz, Iran

[c] Molecular Biology Research Center, Systems Biology and Poisonings Institute, Baqiyatallah University of Medical Sciences, Tehran, Iran

SUPPLEMENTARY INFORMATION

Supplementary Table S1. Cytotoxic T Lymphocyte (CTL) epitopes of the spike protein (S) [a]

Start	End	Length	Peptide	IC_{50}	Rank	Antigenicity	Allergenicity
898	1006	9	FAMQMAYRF	5.03	0.02	1.0278	non-allergen
386	***395***	***10***	***KLNDLCFTNV***	***6.22***	***0.1***	***2.6927***	***non-allergen***
19	27	9	TTRTQLPPA	8.31	0.05	1.254	non-allergen
329	***338***	***10***	***FPNITNLCPF***	***8.63***	***0.02***	***1.3964***	***non-allergen***
200	***209***	***10***	***FKIYSKHTPI***	***9.13***	***0.18***	***1.016***	***non-allergen***
1060	***1068***	***9***	***VVFLHVTYV***	***13.02***	***0.27***	***1.5122***	***non-allergen***
754	763	10	LQYGSFCTQL	13.44	0.06	1.4443	non-allergen
587	***595***	***9***	***ITPCSFGGV***	***20.32***	***0.2***	***1.3871***	***non-allergen***
1207	***1216***	***10***	***EQYIKWPWYI***	***21.01***	***0.08***	***1.1122***	***non-allergen***
265	***273***	***9***	***YYVGYLQPR***	***21.9***	***0.07***	***1.4692***	***non-allergen***
895	904	10	QIPFAMQMAY	23.9	0.1	1.2149	non-allergen
229	***238***	***10***	***LPIGINITRF***	***25.21***	***0.04***	***1.3027***	***non-allergen***
897	906	10	PFAMQMAYRF	28.72	0.1	1.1051	non-allergen
894	904	11	LQIPFAMQMAY	29.34	0.12	1.0701	non-allergen
507	515	9	PYRVVVLSF	30.39	0.11	1.0281	non-allergen
512	***520***	***9***	***VLSFELLHA***	***33.91***	***0.61***	***1.0776***	***non-allergen***
408	***417***	***10***	***RQIAPGQTGK***	***38***	***0.15***	***1.7893***	***non-allergen***
1196	***1205***	***10***	***SLIDLQELGK***	***38.67***	***0.25***	***1.0275***	***non-allergen***
329	337	9	FPNITNLCP	39.11	0.06	1.6218	non-allergen
1059	1068	10	GVVFLHVTYV	39.32	0.69	1.4551	non-allergen
644	***653***	***10***	***QTRAGCLIGA***	***39.78***	***0.34***	***1.3933***	***non-allergen***

[a] Epitopes selected to be used in vaccine polypeptide are shown in bold-italic font.

Supplementary Table S2. Cytotoxic T Lymphocyte (CTL) epitopes of the envelope protein (E) [a]

Start	End	Length	Peptide	IC_{50}	Rank	Antigenicity	Allergenicity
61	***69***	***9***	***RVKNLNSSR***	***4.68***	***0.02***	***0.8998***	***non-allergen***
20	28	9	FLAFVVFLL	5.26	0.04	0.5308	non-allergen
18	***26***	***9***	***LLFLAFVVF***	***8.32***	***0.03***	***0.8144***	***non-allergen***
20	29	10	FLAFVVFLLV	9.95	0.1	0.5651	non-allergen
16	24	9	SVLLFLAFV	11.87	0.15	0.4765	non-allergen
20	29	10	FLAFVVFLLV	12.57	0.26	0.5651	non-allergen
20	28	9	FLAFVVFLL	14.54	0.3	0.5308	non-allergen
57	***65***	***9***	***YVYSRVKNL***	***15.74***	***0.31***	***0.702***	***non-allergen***
30	38	9	TLAILTALR	17.89	0.13	0.7223	non-allergen
17	25	9	VLLFLAFVV	21.72	0.25	0.5677	non-allergen
29	37	9	VTLAILTAL	22.24	0.27	0.614	non-allergen
17	26	10	VLLFLAFVVF	22.52	0.12	0.5362	non-allergen
60	69	10	SRVKNLNSSR	25.59	0.33	0.6612	non-allergen
19	28	10	LFLAFVVFLL	26.16	0.3	0.5111	non-allergen
29	***38***	***10***	***VTLAILTALR***	***26.92***	***0.23***	***0.8404***	***non-allergen***
61	69	9	RVKNLNSSR	27.32	0.14	0.8998	non-allergen
23	***31***	***9***	***FVVFLLVTL***	***27.7***	***0.32***	***0.7403***	***non-allergen***
18	27	10	LLFLAFVVFL	32.72	0.37	0.6159	non-allergen
26	***34***	***9***	***FLLVTLAIL***	***39.95***	***0.43***	***0.9645***	***non-allergen***
15	24	10	NSVLLFLAFV	41.69	0.35	0.4551	non-allergen
20	27	8	FLAFVVFL	42.25	0.45	0.4483	non-allergen
45	***53***	***9***	***NIVNVSLVK***	***46.43***	***0.4***	***0.931***	***non-allergen***

[a] Epitopes selected to be used in vaccine polypeptide are shown in bold-italic font.

Supplementary Table S3. Cytotoxic T Lymphocyte (CTL) epitopes of the membrane protein (M) [a]

Start	End	Length	Peptide	IC_{50}	Rank	Antigenicity	Allergenicity
61	70	10	TLACFVLAAV	3.82	0.04	1.2318	non-allergen
61	69	9	TLACFVLAA	7.63	0.14	1.1932	non-allergen
19	***28***	***10***	***QWNLVIGFLF***	***12.03***	***0.04***	***1.2302***	***non-allergen***
12	***20***	***9***	***IAMACLVGL***	***13.64***	***0.18***	***1.1306***	***non-allergen***
6	***14***	***9***	***GTITVEELK***	***18.08***	***0.13***	***1.0976***	***non-allergen***
35	***44***	***10***	***RTRSMWSFNP***	***18.67***	***0.09***	***1.591***	***non-allergen***
61	70	10	TLACFVLAAV	20.28	0.22	1.2318	non-allergen
6	14	9	GTITVEELK	20.98	0.12	1.0976	non-allergen
60	68	9	VTLACFVLA	24.2	0.29	1.4562	non-allergen
51	***60***	***10***	***SGFAAYSRYR***	***24.22***	***0.31***	***1.0034***	***non-allergen***
22	***30***	***9***	***LVIGFLFLT***	***25.12***	***0.3***	***1.2619***	***non-allergen***
61	70	10	TLACFVLAAV	30.92	0.36	1.2318	non-allergen
26	***34***	***9***	***FLFLTWICL***	***32.26***	***0.36***	***1.4835***	***non-allergen***
61	69	9	TLACFVLAA	33.11	0.37	1.1932	non-allergen
60	69	10	VTLACFVLAA	34.02	0.61	1.3176	non-allergen
10	***18***	***9***	***IAIAMACLV***	***34.16***	***0.39***	***1.1704***	***non-allergen***
60	***70***	***11***	***VTLACFVLAAV***	***40.96***	***0.71***	***1.3368***	***non-allergen***
29	***37***	***9***	***SFRLFARTR***	***41.19***	***0.17***	***0.7038***	***non-allergen***

[a] Epitopes selected to be used in vaccine polypeptide are shown in bold-italic font.

Supplementary Table S4. Cytotoxic T Lymphocyte (CTL) epitopes of the nucleocapsid phosphoprotein (N) [a]

Start	End	Length	Peptide	IC_{50}	Rank	Antigenicity	Allergenicity
305	314	10	AQFAPSASAF	3.58	0.02	0.5986	non-allergen
361	369	9	KTFPPTEPK	6.28	0.02	0.7571	non-allergen
105	113	9	SPRWYFYYL	6.32	0.02	0.734	non-allergen
316	324	9	GMSRIGMEV	6.68	0.11	0.6287	non-allergen
315	***324***	***10***	***FGMSRIGMEV***	***7.16***	***0.13***	***0.88***	***non-allergen***
361	***370***	***10***	***KTFPPTEPKK***	***11.43***	***0.02***	***0.7657***	***non-allergen***
360	369	10	YKTFPPTEPK	11.53	0.07	0.7633	non-allergen
100	***107***	***8***	***KMKDLSPR***	***13.15***	***0.15***	***1.7575***	***non-allergen***
305	315	11	AQFAPSASAFF	22.87	0.12	0.4775	non-allergen
322	331	10	MEVTPSGTWL	28.62	0.09	0.6342	non-allergen
359	369	11	AYKTFPPTEPK	32.64	0.2	0.6958	non-allergen
193	***201***	***9***	***SSRNSTPGS***	***32.96***	***0.16***	***1.2424***	***non-allergen***
78	87	10	SSPDDQIGYY	33.46	0.1	0.4533	non-allergen
104	***113***	***10***	***LSPRWYFYYL***	***37.51***	***0.11***	***1.3486***	***non-allergen***

[a] Epitopes selected to be used in vaccine polypeptide are shown in bold-italic font.

Supplementary Table S5. Final selected helper T lymphocyte epitopes for multi-epitope vaccine construction

Protein	Start	End	Length	Peptide	Core peptide	IC_{50} (nM)	Rank	Antigenicity	IL-10 inducing	IL-4 inducing	IFN-γ inducing	Allergenicity
S	511	525	15	VVLSFELLHAPATVC	FELLHAPAT	2.9	0.03	0.8618	IL-10 inducer	IL-4 inducer	positive	non-allergen
S	166	180	15	CTFEYVSQPFLMDLE	EYVSQPFLM	18.8	0.19	0.57	IL-10 inducer	IL-4 inducer	positive	non-allergen
S	750	764	15	SNLLLQYGSFCTQLN	LLQYGSFCT	10.5	0.27	0.8305	IL-10 inducer	IL-4 inducer	positive	non-allergen
S	168	182	15	FEYVSQPFLMDLEGK	EYVSQPFLM	21.4	0.29	0.8278	IL-10 inducer	IL-4 inducer	positive	non-allergen
S	751	765	15	NLLLQYGSFCTQLNR	LLQYGSFCT	12.1	0.36	0.8668	IL-10 inducer	IL-4 inducer	positive	non-allergen
S	142	156	15	GVYYHKNNKSWMESE	YHKNNKSWM	8.6	0.42	0.4684	IL-10 inducer	IL-4 inducer	positive	non-allergen
S	141	155	15	LGVYYHKNNKSWMES	YHKNNKSWM	9.2	0.48	0.4937	IL-10 inducer	IL-4 inducer	positive	non-allergen
S	1210	1224	15	IKWPWYIWLGFIAGL	YIWLGFIAG	26.9	0.69	0.9153	IL-10 inducer	IL-4 inducer	positive	non-allergen
S	140	154	15	FLGVYYHKNNKSWME	YHKNNKSWM	11.8	0.72	0.4793	IL-10 inducer	IL-4 inducer	positive	non-allergen
S	346	360	15	RFASVYAWNRKRISN	FASVYAWNR	8	0.74	0.4243	IL-10 inducer	IL-4 inducer	positive	non-allergen
S	55	69	15	FLPFFSNVTWFHAIH	FSNVTWFHA	28.8	0.81	0.4883	IL-10 inducer	IL-4 inducer	positive	non-allergen
S	166	180	15	CTFEYVSQPFLMDLE	YVSQPFLMD	32.3	0.97	0.57	IL-10 inducer	IL-4 inducer	positive	non-allergen
N	83	97	15	QIGYYRRATRRIRGG	YRRATRRIR	6.7	0.49	0.4614	IL-10 inducer	IL-4 inducer	positive	non-allergen
N	303	317	15	QIAQFAPSASAFFGM	FAPSASAFF	4.8	0.01	0.4032	IL-10 inducer	IL-4 inducer	positive	non-allergen

Supplementary Table S6. Final selected Linear B lymphocyte epitope for multi-epitope vaccine construction

Protein	Start	End	Peptide	Score	Antigenicity	IL-10 inducing	IL-4 inducing	IFN-γ	Allergenicity
E	48	63	DVSLVKPSFYVYSRVK	0.82	0.8767	IL-10 inducer	IL-4 inducer	positive	non-allergen

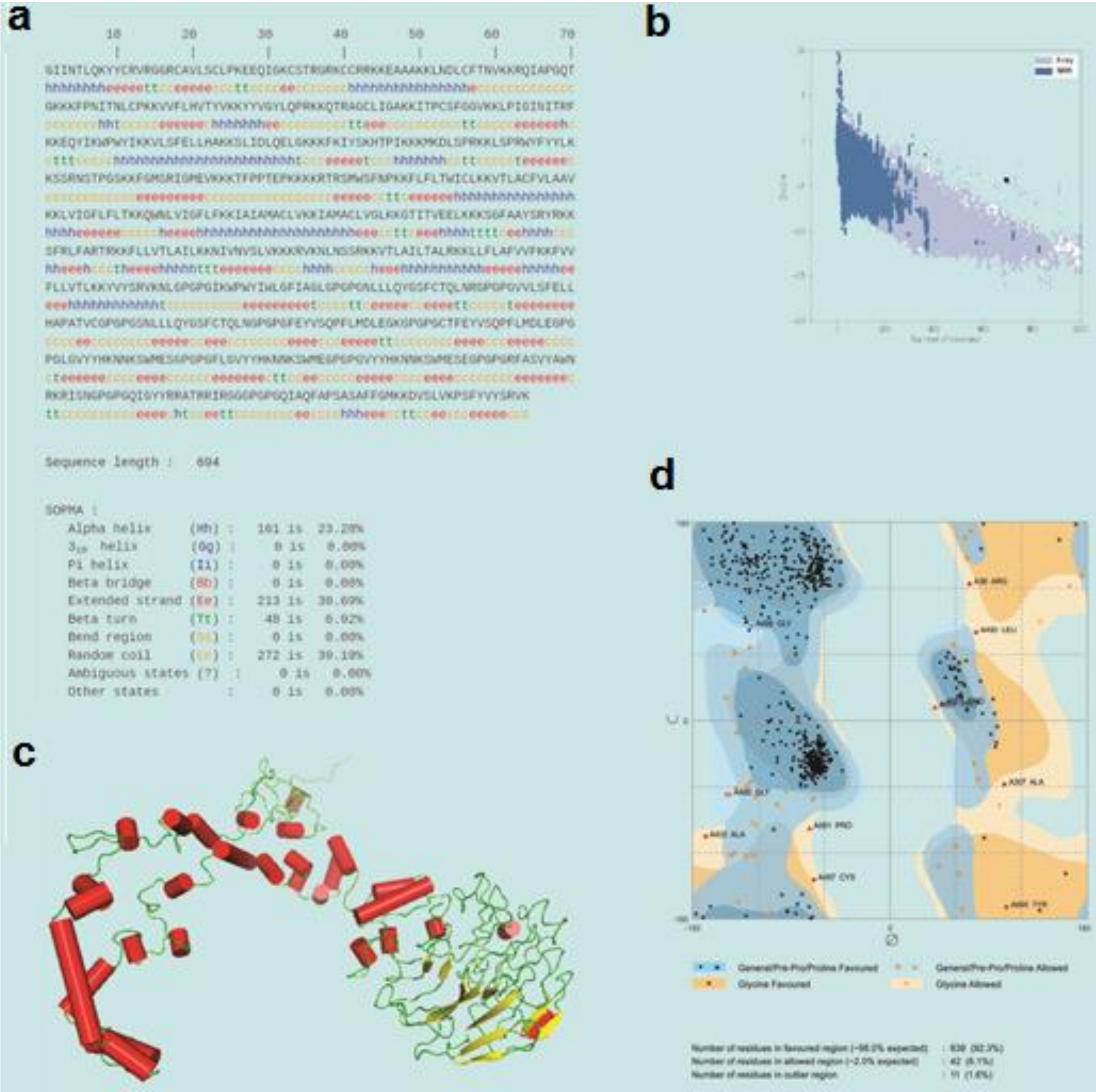

Supplementary Figure S1. Prediction and assessment of the secondary and 3D structure of the multi-epitope vaccine polypeptide. (a) The predicted results showed that among 694 amino acids in the final vaccine construct, 161 (23.20%), 213 (30.69%), 48(6.92%), and 272 (39.19%) amino acids are involved in α-helix, extended strand, β-turn, and random coil, respectively; (b) ProSA validation of 3D vaccine model showing Z-score (-4.33); (c) 3D structure of the vaccine protein; (d) Ramachandran plot analysis of refined structure showing 92.3%, 6.1% and 1.6% residues in favoured, allowed and disallowed regions, respectively.

Supplementary Table S7. Linear and Discontinuous B cell epitopes of the final 3D structure of vaccine

Epitope	Number of residues	Score
**Linear epitopic peptides**		
^{111}AGCLIGAKKITPCSFGGVKKLPIGINITRFKKEQYIKWPWYIKKVLSFELLHAKKSLILQE LGKKKFKIYSKHTPIKKKMKLSPRKKLSPRWYFYYLKKSSRNSTPGSKK^{222}	112	0.878
^{1}GIINTLQKYYCRVRGGRCAVLSCLPKEEQIGKCSTRGRKCCRRKKE^{45}	45	0.869
^{67}PGQTGKKKFPNITN^{80}	14	0.766
^{608}NNKSWME^{614}	7	0.737
^{566}YYHKNNKSWMESGPGP^{581}	16	0.735
^{225}MSRI^{228}	4	0.728
^{529}PFLMLEGKG^{538}	10	0.726
^{54}DLCF^{57}	4	0.713
^{511}SFCT^{514}	4	0.7
**Residues from discontinuous epitopes**		
G112, C113, L114, I115, G116, A117, K118, K119, I120, T121, P122, C123, S124, F125, G126, G127, V128, K129, K130, L131, P132, I133, G134, I135, N136, I137, T138, R139, F140, K141, E143, Q144, Y145, I146, K147, W148, P149, W150, Y151, I152, K153, K154, V155, L156, S157, F158, E159, L160, L161, H162, A163, K164, K165, S166, L167, I168, D169, L170, Q171, E172, L173, G174, K175, K176, K177, F178, K179, I180, Y181, S182, K183, H184, T185, P186, I187, K188, K189, K190, M191, K192, D193, L194, S195, P196, R197, K198, L200, S201, P202, R203, W204, Y205, F206, Y207, Y208, L209, K210, K211, S212, S213, R214, N215, S216, T217, P218, G219, S220, K222	108	0.882
G1, I2, I3, N4, T5, L6, Q7, K8, Y9, Y10, C11, R12, R14, G15, G16, R17, C18, A19, V20, L21, S22, C23, L24, P25, K26, E27, E28, Q29, I30, G31, K32, C33, S34, T35, R36, G37, R38, K39, C40, C41, R43, K50, K51, A66, P67, G68, Q69, T70, G71, K72, K73, K74	52	0.847
N608, N609, K610, S611, W612, M613	6	0.74
M225, S226, R227, I228	4	0.728
D54, L55, C56, F57, T58, F75, P76, N77, I78, T79, N80, L81, C82	13	0.709
Q528, P529, F530, L531, M532, L534, E535, G536, K537, G538, P539	11	0.709
Y509, G510, S511, C513, T514, V548, S549, Q550, P551, F552, M554, D555, L556, G562, L563, G564, V565, Y566, Y567, H568, K569, N570, N571, K572, S573, W574, M575, E576, S577, G578, P579, G580, P581	33	0.702

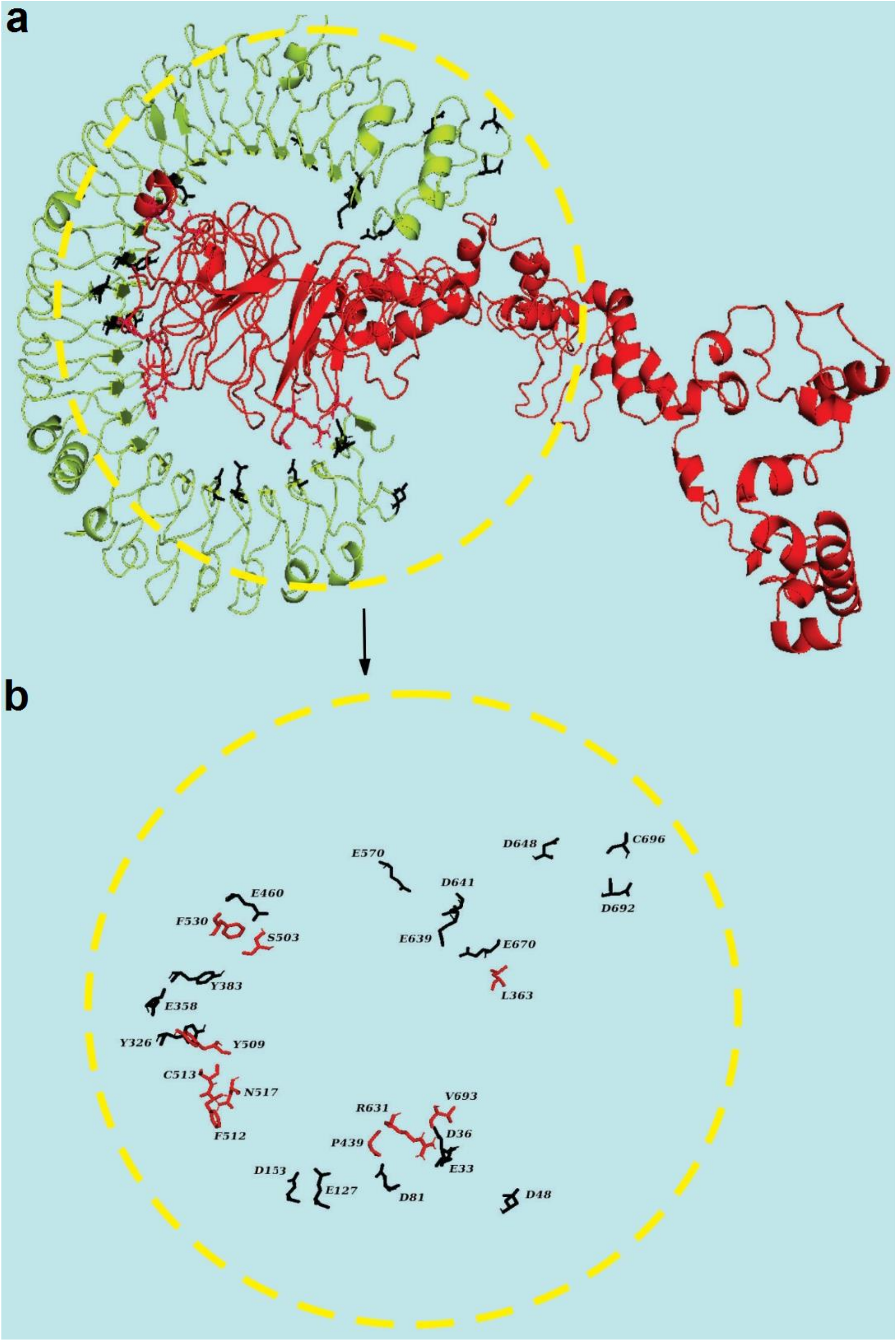

Supplementary Figure S2. Illustration of the interaction between the designed vaccine polypeptide and the immune receptor Toll-like receptor-3 (TLR-3) as shown by molecular docking. (a) Docked complex of TLR-3 (PDB ID: 2A0Z) (chartreuse colour) with the multi-epitope vaccine (red colour); (b) Residues contributing in the vaccine polypeptide-receptor interaction.

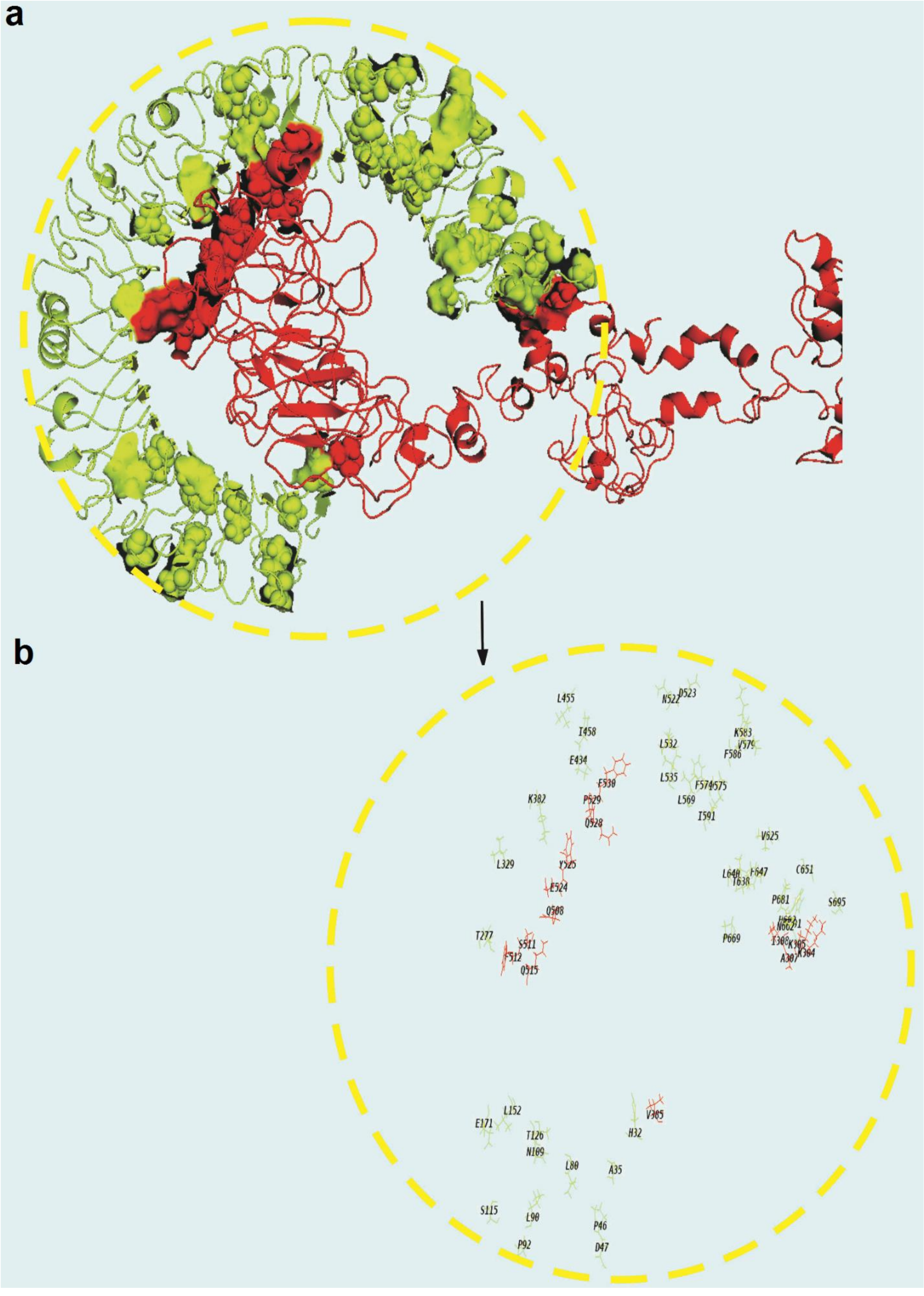
a
b
L455
N522
D523
I458
K583
L532
F586
E434
F530
L535
L569
K382
P529
Q528
I591
V625
L329
F647
C651
E524
P681
S695
Q508
P669
T277
S511
Q515
L152
V305
E171
T126
H32
N109
L80
A35
S115
L90
P46
P92
D47

Supplementary Figure S3. Illustration of the interaction between the vaccine polypeptide and the immune receptor Toll-like receptor-3 (TLR-3). (a) Arrangement of the vaccine protein residues (red spheres) in the binding pocket of TLR-3 (chartreuse spheres); (b) The key residues in the binding free energy. Vaccine polypeptide residues are shown in red, Toll-like receptor-3 residues in chartreuse.

Supplementary Table S8. HLAs class I and class II which were used in the prediction of CTL and HTL epitopes

**HLAs class I**			**HLAs class II**
HLA-A*01:01,8	HLA-A*30:01,11	HLA-B*15:01,10	HLA-DRB1*01:01
HLA-A*01:01,9	HLA-A*30:02,8	HLA-B*15:01,11	HLA-DRB1*03:01
HLA-A*01:01,10	HLA-A*30:02,9	HLA-B*35:01,8	HLA-DRB1*04:01
HLA-A*01:01,11	HLA-A*30:02,10	HLA-B*35:01,9	HLA-DRB1*04:05
HLA-A*02:01,8	HLA-A*30:02,11	HLA-B*35:01,10	HLA-DRB1*07:01
HLA-A*02:01,9	HLA-A*31:01,8	HLA-B*35:01,11	HLA-DRB1*08:02
HLA-A*02:01,10	HLA-A*31:01,9	HLA-B*40:01,8	HLA-DRB1*09:01
HLA-A*02:01,11	HLA-A*31:01,10	HLA-B*40:01,9	HLA-DRB1*11:01
HLA-A*02:03,8	HLA-A*31:01,11	HLA-B*40:01,10	HLA-DRB1*12:01
HLA-A*02:03,9	HLA-A*32:01,8	HLA-B*40:01,11	HLA-DRB1*13:02
HLA-A*02:03,10	HLA-A*32:01,9	HLA-B*44:02,8	HLA-DRB1*15:01
HLA-A*02:03,11	HLA-B*58:01,8	HLA-B*44:02,9	HLA-DRB3*01:01
HLA-A*02:06,8	HLA-B*58:01,9	HLA-B*44:02,10	HLA-DRB3*02:02
HLA-A*02:06,9	HLA-B*58:01,10	HLA-B*44:02,11	HLA-DRB4*01:01
HLA-A*02:06,10	HLA-B*58:01,11	HLA-B*44:03,8	HLA-DRB5*01:01
HLA-A*02:06,11	HLA-A*32:01,10	HLA-B*44:03,9	HLA-DQA1*05:01/DQB1*02:01
HLA-A*03:01,8	HLA-A*32:01,11	HLA-B*44:03,10	HLA-DQA1*05:01/DQB1*03:01
HLA-A*03:01,9	HLA-A*33:01,8	HLA-B*44:03,11	HLA-DQA1*03:01/DQB1*03:02
HLA-A*03:01,10	HLA-A*33:01,9	HLA-B*51:01,8	HLA-DQA1*04:01/DQB1*04:02
HLA-A*03:01,11	HLA-A*33:01,10	HLA-B*51:01,9	HLA-DQA1*01:01/DQB1*05:01
HLA-A*11:01,8	HLA-A*33:01,11	HLA-B*51:01,10	HLA-DQA1*01:02/DQB1*06:02
HLA-A*11:01,9	HLA-A*68:01,8	HLA-B*51:01,11	HLA-DPA1*02:01/DPB1*01:01
HLA-A*11:01,10	HLA-A*68:01,9	HLA-B*53:01,8	HLA-DPA1*01:03/DPB1*02:01
HLA-A*11:01,11	HLA-A*68:01,10	HLA-B*53:01,9	HLA-DPA1*01:03/DPB1*04:01
HLA-A*23:01,8	HLA-A*68:01,11	HLA-B*53:01,10	HLA-DPA1*03:01/DPB1*04:02
HLA-A*23:01,9	HLA-A*68:02,8	HLA-B*53:01,11	HLA-DPA1*02:01/DPB1*05:01
HLA-A*23:01,10	HLA-A*68:02,9	HLA-B*57:01,8	HLA-DPA1*02:01/DPB1*14:01
HLA-A*23:01,11	HLA-A*68:02,10	HLA-B*57:01,9	
HLA-A*24:02,8	HLA-A*68:02,11	HLA-B*57:01,10	
HLA-A*24:02,9	HLA-B*07:02,8	HLA-B*57:01,11	
HLA-A*24:02,10	HLA-B*07:02,9		
HLA-A*24:02,11	HLA-B*07:02,10		
HLA-A*26:01,8	HLA-B*07:02,11		
HLA-A*26:01,9	HLA-B*08:01,8		
HLA-A*26:01,10	HLA-B*08:01,9		
HLA-A*26:01,11	HLA-B*08:01,10		
HLA-A*30:01,8	HLA-B*08:01,11		
HLA-A*30:01,9	HLA-B*15:01,8		
HLA-A*30:01,10	HLA-B*15:01,9		
